# Stochastic modeling, analysis, and simulation of the COVID-19 pandemic with explicit behavioral changes in Bogotá: A case study

**DOI:** 10.1016/j.idm.2021.12.008

**Published:** 2021-12-31

**Authors:** David Niño-Torres, Andrés Ríos-Gutiérrez, Viswanathan Arunachalam, Comfort Ohajunwa, Padmanabhan Seshaiyer

**Affiliations:** a Department of Statistics, Universidad Nacional de Colombia, Bogotá, Colombia; b George Mason University, Fairfax, VA, USA

**Keywords:** Epidemic spread, SARS-CoV-2, Compartmental modeling, Stochastic perturbations, Parameter estimation

## Abstract

In this paper, a stochastic epidemiological model is presented as an extension of a compartmental SEIR model with random perturbations to analyze the dynamics of the COVID-19 pandemic in the city of Bogotá D.C., Colombia. This model incorporates the spread of COVID-19 impacted by social behaviors in the population and allows for projecting the number of infected, recovered, and deceased individuals considering the mitigation measures, namely confinement and partial relaxed restrictions. Also, the role of randomness using the concept of Brownian motion is emphasized to explain the behavior of the population. Computational experiments for the stochastic model with random perturbations were performed, and the model is validated through numerical simulations for actual data from Bogotá D.C.

## 1 Introduction

The coronavirus disease (COVID-19) that is believed to have emerged in Wuhan, China, in late 2019 spread to Colombia on March 6, 2020, when the country had reported its first positive case (Benıétezet al., 2020; Ministerio de Salud y Protección Social, 2020). The same month, the World Health Organization had declared the disease as a pandemic (World Health Organization, 2020). These developments have prompted much research dedicated to understanding the nature of the spread of the disease (Sohrabiet al., 2020; Wanget al., 2020; Wu et al., 2020). Specifically, various mathematical models have been widely employed to predict the future spread of the disease as well as explore the impact of certain interventions on the spread(see (Amouch & Karim, 2021; Linet al., 2020; Musaet al., 2021)).

Most mathematical models describing the spread of the disease employ classical compartments, such as the Susceptible-Exposed-Infected-Recovered (SEIR) structure described as an ordinary differential equation system (Brauer and Castillo-Chavez, 2012). Some researchers have adapted these models to include several relevant factors, including the role of containment strategies (Maier and Brockmann, 2020), mobility patterns (Rădulescu et al., 2020), social distancing (Leunget al., 2018; Premet al., 2020), and network intervention strategies to patterns of plateau duration, intensity and duration of social distancing measures (Komarova et al., 2021).

However, the pandemic has prompted lock-downs, widespread closures, and calls to social distance and practice basic hygiene, which has encouraged many people to take caution to limit the spread (Alberto and GongGiordano, 2021; Orabyet al., 2021). To account for the impact of such non-pharmaceutical interventions and the social behavior of the population in response, recently a novel mathematical framework was developed (Ohajunwa et al., 2020). Specifically, three separate models including a baseline model, an explicit intervention model, and an implicit intervention model were created. Of particular relevance to this paper is the explicit intervention model, which accounted for the effect of lock-down through the addition of a Confined compartment to an extended SEIR model. Based on Dirac delta functions, a portion of susceptible individuals was modeled to enter confinement during lock-down and return to become susceptible when lock-down ended. While this model was able to incorporate social behavior through the transmission parameters, this model did not include the influence of stochasticity. Given that many individuals within the population are assumed to react irrationally and unpredictably in response to the pandemic, there is a need to consider stochastic models to capture these effects. Stochastic perturbation models can be used to study the effects of these reactions by introducing environmental noise on the transmission parameter (see (Cai et al., 2019; Grayet al., 2011; Ríos-Gutiérrez et al., 2021)).

Colombia, like many other countries, has implemented a series of emergency measures in response to the coronavirus outbreak. The government quickly responded by policy implementation by introducing strict lock-downs, PCR testing capacity, contact tracing, and augmenting ICU capacity in the hospitals. While Colombia's management of COVID-19 may be considered as a success, the country was not been able to flatten the curve for more than 100 days at the beginning of the pandemic (see (Cárdenas & Martínez, 2020; De la Hoz-Restrepoet al., 2020)). Detailed daily reports and other information can be found at (Instituto Nacional de Salud de Colombia; Ministerio de Salud y Protección Social, 2020).

Thus, the goal of this paper is to develop a novel mathematical framework that extends the previous model (Ohajunwa et al., 2020) to not only account for the effects of social behavior, specifically during lock-down conditions, but also the role of stochasticity within the population. Furthermore, we also apply this model to real data from the Colombian city of Bogotá through computational techniques. Specifically, we use data from the National Institute of Health of Colombia (see (Instituto Nacional de Salud de Colombia)) to derive parameters for our simulations and analysis. This model will project the number of infected, recovered and deceased individuals taking into account not only mitigation measures, specifically confinement and partial opening, but also the role of randomness in the behavior of the population in the pandemic.

This paper will be structured as follows. In section 2, we introduce the stochastic ordinary differential equation system, including the definition of the variables and parameters. The section also describes the numerical discretization for this system along with calibration of the model. In section 3, we validate the model by performing numerical simulations against real data from Bogotá. Finally, in section 4, we discuss and draw conclusions from our work.

## 2 A stochastic model: equations and methods

To address the disease dynamics of the COVID-19 pandemic in the city of Bogotá DC, we propose a stochastic compartmental disease transmission model based on (Ohajunwa et al., 2020) and adapted according to a structure of stochastic differential equations. The phases or compartments are based on the scientific literature and the different decrees adopted by the various governmental and health institutions in charge.

**Fig. 1:**
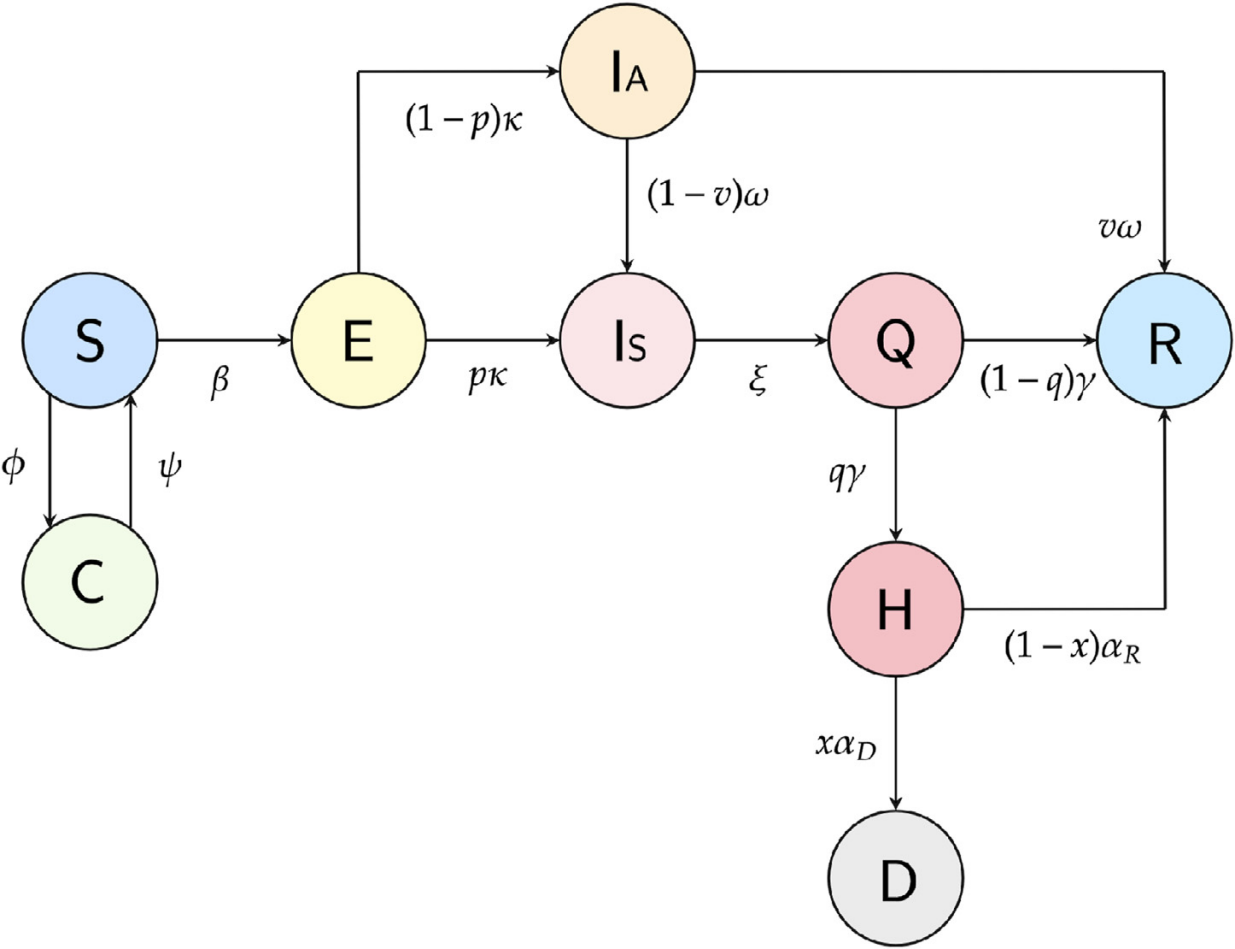
A flow diagram of the proposed model dynamics.

The flow diagram of the proposed mathematical model is shown in Fig. 1. Specifically, individuals transition from one compartment to another according to specific rates and are influenced by noise, which simulates the random dynamics of the epidemic. The model assumes that the total population is homogeneous and constant in size *N*. Immigration and emigration are not taken into account; that is, the population is closed.

For this work, we introduce the following the sub-populations: Susceptible (*S*), Confined (*C*), Exposed (*E*), Asymptomatic (*I*_*A*_), Symptomatic (*I*_*S*_), Quarantined (*Q*), Hospitalized (*H*), Recovered (*R*), and Dead (*D*). Susceptible individuals consist of individuals who are not infected with COVID-19 and are not isolated from the population. Confined individuals are individuals who were previously susceptible but by their own or regulatory decision are temporarily isolated from the population in order to protect themselves from infection. Exposed individuals are individuals who are no longer susceptible because they have come into contact with asymptomatic or symptomatic infected individuals and are in the incubation period of disease progression. Asymptomatic individuals are infected individuals who do not exhibit any symptoms of COVID-19, while symptomatic individuals are infected individuals who do have symptoms. Hospitalized individuals are individuals who have been symptomatic and enter hospitalization with severe COVID-19 symptoms. Quarantined individuals are symptomatic individuals who have been isolated. Note that in this model, we suppose that symptomatic population immediately go to quarantine and does not include any death of this population. However, with more available data, we could consider this case in the future. Recovered individuals consist of individuals who were previously infectious and has now survived COVID-19. Deceased individuals are individuals who were infectious but did not survive COVID-19. Given that, we assume a closed population size, *N* = *S* + *C* + *E* + *I*_*A*_ + *I*_*S*_ + *H* + *Q* + *R* + *D*.

In addition, we define the parameters used for the model in Table 1. The rates at which susceptible and confined individuals enter and leave confinement depend on the Dirac delta functions *φ*(*t*) and *ψ*(*t*). Susceptible individuals become exposed upon contact with the infected population at the transmission rate *β*. Exposed individuals become infected at the incubation rate, *κ*, such that a fraction *p* becomes symptomatic infectious and the remaining (1 − *p*) becomes asymptomatic infectious. Asymptomatic individuals either recover or develop symptoms at a rate of *ω*, with a fraction *ν* recovering and a fraction (1 − *ν*) becoming symptomatic. Symptomatic individuals quarantine at a rate of *ξ*. At a rate of *γ*, a fraction *q* of quarantined individuals are hospitalized and a fraction (1 − *q*) recover. Lastly, a proportion *x* of hospitalized individuals die at a rate of *α*^*D*^, while a proportion (1 − *x*) of hospitalized individuals recover at a rate of *α*^*R*^.

**Table 1:** Definition of Parameters in the model.

Parameter	Definition
*β*	Transmission Rate
*φ*	Rate at which susceptible individuals enter confinement as a function of time
*ψ*	Rate at which confined individuals reenter the susceptible sub-population as a function of time
*κ*	Incubation rate
*ω*	Rate at which asymptomatic individuals become symptomatic or recover
*ξ*	Rate at which symptomatic individuals enter quarantine
*γ*	Rate at which quarantined individuals become hospitalized or recover
*α*_*R*_	Recovery rate for hospitalized individuals
*α*_*D*_	Death rate for hospitalized individuals
*p*	Proportion of exposed individuals that become symptomatic
*q*	Proportion of quarantined individuals that become hospitalized
*ν*	Proportion of asymptomatic individuals that recover
*x*	Proportion of hospitalized individuals that die
*l*	Proportion of susceptible individuals that enter confinement at *t* = *t*_*m*_
*m*	Proportion of confined individuals that reenter the susceptible population at *t* = *t*_*l*_
*t*_*l*_	Lock-down release time (in days from *t* = 0)
*t*_*m*_	Lock-down time (in days from *t* = 0)

Next, we propose the following system of stochastic differential equations for the *modified Stochastic SEIR model with random perturbations* (see (Ríos-Gutiérrez et al., 2021)). Note that this model will include random perturbations of the white noise terms satisfying the Brownian motion governed by system of stochastic differential equations. Let ${\left\{S\left(t\right),E\left(t\right),I^{A}\left(t\right),I^{S}\left(t\right),Q\left(t\right),H\left(t\right),R\left(t\right),D\left(t\right),C\left(t\right)\right\}}_{t\geq 0}$ be an Itô process given by the following system of differential equations:

(1) $$\begin{array}{ccc} dS\left(t\right) & = & \left[-\varphi S\left(t\right)+\psi C\left(t\right)-S\left(t\right)\tilde{\beta }\left(\frac{I^{A}\left(t\right)+I^{S}\left(t\right)}{N}\right)\right]dt-\frac{\sigma_{1}S\left(t\right)I^{A}\left(t\right)}{N}dB_{1}\left(t\right)-\frac{\sigma_{2}S\left(t\right)I^{S}\left(t\right)}{N}dB_{2}\left(t\right)\\ \frac{dE\left(t\right)}{dt} & = & \tilde{\beta }S\left(t\right)\left(\frac{I^{A}\left(t\right)+I^{S}\left(t\right)}{N}\right)-\kappa E\left(t\right)\\ {\mathrm{dI}}^{A}\left(t\right) & = & \left(\left(1-p\right)\kappa E\left(t\right)-\omega I^{A}\left(t\right)\right)dt+\frac{\sigma_{1}S\left(t\right)I^{A}\left(t\right)}{N}dB_{1}\left(t\right)\\ {\mathrm{dI}}^{S}\left(t\right) & = & \left(p\kappa E+\left(1-\nu \right)\omega I^{A}\left(t\right)-\xi I^{S}\left(t\right)\right)dt+\frac{\sigma_{2}S\left(t\right)I^{S}\left(t\right)}{N}dB_{2}\left(t\right)\\ \frac{dQ\left(t\right)}{dt} & = & \xi I^{S}\left(t\right)-\gamma Q\left(t\right)\\ \frac{dH\left(t\right)}{dt} & = & q\gamma Q\left(t\right)-\left(\left(1-x\right)\alpha_{R}+x\alpha_{D}\right)H\left(t\right)\\ \frac{dR\left(t\right)}{dt} & = & \nu \omega I^{A}\left(t\right)+\left(1-q\right)\gamma Q\left(t\right)+\left(1-x\right)\alpha_{R}H\left(t\right)\\ \frac{dD\left(t\right)}{dt} & = & x\alpha_{D}H\left(t\right)\\ \frac{dC\left(t\right)}{dt} & = & \varphi S\left(t\right)-\psi C\left(t\right), \end{array}$$

where ${\left\{B_{1}\left(t\right)\right\}}_{t\geq 0}$ and ${\left\{B_{2}\left(t\right)\right\}}_{t\geq 0}$ are two independent standard Brownian motions, and considered as generalized white noise functionals, and the constants *σ*_1_ and *σ*_2_ are the intensities of the noises. We note that ${\left\{B_{1}\left(t\right)\right\}}_{t\geq 0}$ and ${\left\{B_{2}\left(t\right)\right\}}_{t\geq 0}$ are defined on the complete probability space $\left(\Omega ,I,{\left\{I\right\}}_{t\geq 0},P\right)$ satisfying the usual conditions . The functions *φ* = *φ*(*t*) = *lδ*(*t* − *t*_*l*_) and *ψ* = *ψ*(*t*) = *mδ*(*t* − *t*_*m*_), such that:

(2) $$\delta \left(t-t_{x}\right)=\left\{\begin{array}{ll} 1\; & \text{ if }t-t_{x}=0\\ 0\; & \text{ if }t-t_{x}\neq 0 \end{array}\right)$$

Since the transmission rate changes according to the dynamics of the confinement or lock, then the following is proposed:

(3) $$\tilde{\beta }\left(t-t_{l}\right)=\left\{\begin{array}{ll} \left(1+\varepsilon \right)\beta \; & \text{ if }t-t_{l}>0\\ \beta \; & \text{ if }t-t_{l}\leq 0. \end{array}\right)$$

### 2.1 Basic reproduction number

We now briefly discuss the basic reproduction number *R*_0_, which is the expected number of secondary cases produced by a single infection in a completely susceptible population. Using the method for the epidemic models with random perturbations (Ríos-Gutiérrez et al., 2021), we define the basic reproduction number as follows:

(4) $$R_{0}≔\underset{0}{\overset{\infty }{\int }}b\left(a\right)F\left(a\right)da\text{,}$$

where $b\left(a\right)$ is the average number of newly infected individuals (in a completely susceptible population) by an infected individual that is infectious between *t* = 0 and *t* = *a*. $F\left(a\right)$ is the probability that a newly infected individual will continue infecting others during the time interval between 0 and *a*. This is also called the underlying survival probability (or survival function).

In our case, the infectivity rate when the population is completely susceptible is given by

$${\tilde{\beta }}_{1}≔\frac{\beta }{N}+\frac{\sigma_{1}}{N}B_{1}\left(t\right)$$

if the first infected individual is asymptomatic, or

$${\tilde{\beta }}_{2}=\frac{\beta }{N}+\frac{\sigma_{2}}{N}B_{2}\left(t\right)$$

if the first infected individual is symptomatic. When the first infected individual infects a completely susceptible population with *N* individuals, then the expected number of asymptomatic individuals who were infected by a symptomatic individual is

$$b_{1}\left(a\right)=N{\tilde{\beta }}_{2}\left(1-p\right)=\left(\beta +\sigma_{2}E\left(B_{2}\left(+\infty \right)\right)\right)\left(1-p\right),$$

the expected number of symptomatic individuals who were infected by a symptomatic individual is

$$b_{2}\left(a\right)=N{\tilde{\beta }}_{2}p=\beta +\sigma_{2}E\left(B_{2}\left(+\infty \right)\right)p,$$

and the expected number of asymptomatic individuals who were infected by a asymptomatic individual and have not recovered from the illness, that is, became in symptomatic individuals is

$$b_{3}\left(a\right)=N{\tilde{\beta }}_{1}\left(1-\upsilon \right)\left(1-p\right)=\left(\beta +\sigma_{1}E\left(B_{1}\left(+\infty \right)\right)\right)\left(1-\upsilon \right)\left(1-p\right),$$

and the expected number of symptomatic individuals who were infected by an asymptomatic individual is

$$b_{4}\left(a\right)=N{\tilde{\beta }}_{1}p=\beta +\sigma_{1}E\left(B_{1}\left(+\infty \right)\right)p.$$

Note that we wrote *E*(*B*_*i*_(+*∞*)) = lim_*t*→+*∞*_*B*_*i*_(*t*) = 1, 2 due to the need to establish how many people are infected by an infectious individual, which we can interpret as the time of infecting from the beginning to the end of time.

In this way.

(*β* + *σ*_2_*B*_2_(*a*))*p* + (*β* + *σ*_2_*B*_2_(*a*))(1 − *p*) + (*β* + *σ*_1_*B*_1_(*a*))(1 − *υ*)(1 − *p*) + (*β* + *σ*_1_*B*_1_(*a*))*p*

is the number of infected individuals by an infectious individual for *a* → + *∞*. We have the survival functions, *F*_1_(*a*) = *e*^−*ξa*^ and *F*_2_(*a*) = *e*^−*ωa*^, for when (1) a symptomatic individual infected by a symptomatic individual remains symptomatic on [0, *a*], (2) an asymptomatic individual infected by a symptomatic individual remains asymptomatic on [0, *a*] and (3) an asymptomatic individual infected by an asymptomatic individual remains infectious on [0, *a*], and (4) a symptomatic individual infected by an asymptomatic individual remains symptomatic on [0, *a*]; respectively. We obtain the following equation

$$\begin{array}{lll} R_{0}^{\text{Cov}} & ≔ & \underset{0}{\int^{+\infty }}b\left(a\right)F\left(a\right)da\\ & = & \underset{0}{\int^{+\infty }}\beta pF_{1}\left(a\right)da+\underset{0}{\int^{+\infty }}\beta \left(1-p\right)F_{2}\left(a\right)da+\underset{0}{\int^{+\infty }}\beta \left(1-\upsilon \right)\left(1-p\right)F_{1}\left(a\right)da+\underset{0}{\int^{+\infty }}\beta pF_{1}\left(a\right)da \end{array}$$

Therefore, in the following equation we introduce *b*_*e*_(*a*) and *F*_*e*_(*a*) which are functions corresponding to *b*(*a*) and *F*(*a*), respectively, for the stochastic system (1). We then have,

(5) $$\underset{0}{\int^{+\infty }}b_{e}\left(a\right)F_{e}\left(a\right)da=2\frac{\beta p}{\xi }+\frac{\beta (1-p)}{\omega }+\frac{\beta (1-\upsilon )(1-p)}{\xi }+\underset{0}{\int^{+\infty }}\sigma_{2}B_{2}\left(a\right)\left(pe^{-\xi a}+\left(1-p\right)e^{-\omega a}\right)da+\underset{0}{\int^{+\infty }}\sigma_{1}\left(\left(1-\upsilon \right)\left(1-p\right)+p\right)B_{1}\left(a\right)e^{-\xi a}da.$$

The integral $\int_{0}^{+\infty }b_{e}\left(a\right)F_{e}\left(a\right)da$ is the mean of the random variable that describes the basic reproduction number for the proposed stochastic model. Based on (Ríos-Gutiérrez et al., 2021), we have that

(6) $$R_{1}≔\underset{0}{\int^{+\infty }}\sigma_{1}\left(\left(1-\upsilon \right)\left(1-p\right)+p\right)B_{1}\left(a\right)e^{-\xi a}da\sim N\left(0,\frac{\sigma_{1}^{2}{\left({\left(1-\upsilon \right)}^{2}(1-p)+p\right)}^{2}}{2\xi^{3}}\right)$$

On the other hand, using integration-by-parts rule (Chung and Williams, 2014), we have that

$$\underset{l\to +\infty }{\lim\;}B_{2}\left(l\right)\left(\omega pe^{-\xi l}+\xi \left(1-p\right)e^{-\omega l}\right)=B_{2}\left(0\right)\left(p\omega +\left(1-p\right)\xi \right)-I_{1}-I_{2}+I_{3}$$

where,

$$\begin{array}{lll} I_{1} & = & \underset{0}{\int^{+\infty }}\omega p\xi B_{2}\left(a\right)e^{-\xi a}da\\ I_{2} & = & \underset{0}{\int^{+\infty }}\xi \left(1-p\right)\omega B_{2}\left(a\right)e^{-\omega a}da\\ I_{3} & = & \underset{0}{\int^{+\infty }}\left(\omega pe^{-\xi a}+\xi \left(1-p\right)e^{-\omega a}\right)dB_{2}\left(a\right)\text{,} \end{array}$$

We compute that lim_*l*→+*∞*_*B*_2_(*l*)(*ωpe*^−*ξl*^ + *ξ*(1 − *p*)*e*^−*ωl*^) = 0 a.s. In addition, *B*_2_(0) = 0 a.s. Now using (Klebaner, 2012)[p. 393], we obtain

$$\begin{array}{lll} R_{2} & ≔ & \underset{0}{\int^{+\infty }}\sigma_{2}B_{2}\left(a\right)\left(pe^{-\xi a}+\left(1-p\right)e^{-\omega a}\right)da\\ & = & \underset{0}{\int^{+\infty }}\sigma_{2}\left(\omega pe^{-\xi a}+\xi \left(1-p\right)e^{-\omega a}\right)dB_{2}\left(a\right)\\ & \sim & N\left(0,\frac{\sigma_{2}^{2}{\left(1-p\right)}^{2}}{2\omega^{3}}+\frac{2\sigma_{2}^{2}p(1-p)}{(\xi +\omega )\xi \omega }\right) \end{array}$$

since we assumed ${\left\{B_{1}\left(t\right)\right\}}_{t\geq 0}$ and ${\left\{B_{2}\left(t\right)\right\}}_{t\geq 0}$ are two Brownian independent motions, in consequence, we get

(7) $$R_{0,v}^{\text{Cov}}=\underset{0}{\overset{+\infty }{\int }}b_{e}\left(a\right)F_{e}\left(a\right)da=R_{0}^{\text{Cov}}+R_{2}+R_{1}∼N\left(R_{0}^{\text{Cov}},Var\left(R_{1}\right)+Var\left(R_{2}\right)\right).$$

Thus, we obtain the distribution of the basic reproduction number for the proposed model.

### 2.2 Numerical discretization

We now briefly explain the numerical approximation method used in the paper. The Euler-Maruyama method described in (Kloeden and Platen, 1992) and introduced by the Japanese mathematician G. Maruyama (Maruyama, 1955) as an extension of the Euler method, is a numerical integration technique for obtaining approximate solutions for a system of stochastic differential equations from a given initial value *X*_0_ = *x*_0_. Let 0 = *t*_0_ < *t*_1_ < ⋯ < *t*_*k*−1_ < *t*_*k*_ = *T* be a partition of the interval [0, *T*], where the length of each subinterval is Δ*t* = *t*_*i*+1_ − *t*_*i*_ = *T*/*k*, which implies that *t*_*i*+1_ = *t*_*i*_ + Δ*t* = *i*Δ*t* and Δ*B*_*i*_ = Δ*B*(*t*_*i*_) = *B*(*t*_*i*_ + Δ*t*) − *B*(*t*_*i*_). For each stochastic process trajectory, the value of $X_{t_{i+1}}$ is approximated using only the value of the previous time step, $X_{t_{i}}$. Then, to find the trajectories or approximate solutions of a stochastic differential equation by the Euler-Maruyama method, the following equation is implemented:

(8) $$X_{t_{i+1}}=X_{t_{i}}+\mu \left(t_{i},X_{t_{i}}\right)\Delta t+\sigma \left(t_{i},X_{t_{i}}\right)\Delta B_{i}$$

for all *i* = 0, 1, …, *k* − 1. In order to carry out the method computationally, it is necessary to know how to calculate Δ*B*_*i*_. Since the partition is made up of equal intervals, the differences Δ*B*_*i*_, *i* = 0, 1, …, *k* − 1 have the same distribution, Δ*B*_*i*_ ∼ *N*(0, Δ*t*). Let *η* be a random variable with a standard normal distribution *η* ∼ *N*(0, 1). Then $\sqrt{\Delta t}\eta$ has a normal distribution with zero mean and variance Δ*t*; that is, $\sqrt{\Delta t}\eta ∼N\left(0,\Delta t\right)$. We use the following algorithm to implement the Euler-Maruyama method to find the approximate solutions of the stochastic differential equations:

**Figure:**
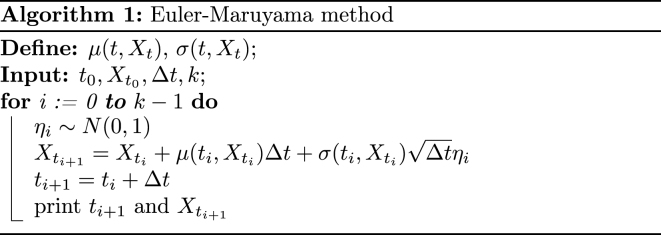

To implement in an analogous way the algorithm of the Euler-Maruyama method, previously described, for our proposed model, the respective discretization of the system of stochastic differential equation (1) must be carried out, which is given by:

(9) $$\begin{array}{ccc} S_{t_{i+1}} & = & S_{t_{i}}-\left[\varphi S_{t_{i}}-\psi C_{t_{i}}+\beta S_{t_{i}}\left(\frac{I_{t_{i}}^{A}+I_{t_{i}}^{S}}{N}\right)\right]\Delta t-\frac{S_{t_{i}}\sigma_{1}I_{t_{i}}^{A}}{N}\sqrt{\Delta t}\eta_{1_{i}}-\frac{S_{t_{i}}\sigma_{2}I_{t_{i}}^{S}}{N}\sqrt{\Delta t}\eta_{2_{i}}\\ C_{t_{i+1}} & = & C_{t_{i}}+\left[\varphi S_{t_{i}}-\psi C_{t_{i}}\right]\Delta t\\ E_{t_{i+1}} & = & E_{t_{i}}+\left[\beta S_{t_{i}}\left(\frac{I_{t_{i}}^{A}+I_{t_{i}}^{S}}{N}\right)-\kappa E_{t_{i}}\right]\Delta t\\ I_{t_{i+1}}^{A} & = & I_{t_{i}}^{A}+\left[\left(1-p\right)\kappa E_{t_{i}}-\omega I_{t_{i}}^{A}\right]\Delta t+\frac{S_{t_{i}}\sigma_{1}I_{t_{i}}^{A}}{N}\sqrt{\Delta t}\eta_{1_{i}}\\ I_{t_{i+1}}^{S} & = & I_{t_{i}}^{S}+\left[p\kappa E_{t_{i}}+\left(1-\nu \right)\omega I_{t_{i}}^{A}-\xi I_{t_{i}}^{S}\right]\Delta t+\frac{S_{t_{i}}\sigma_{2}I_{t_{i}}^{S}}{N}\sqrt{\Delta t}\eta_{2_{i}}\\ Q_{t_{i+1}} & = & Q_{t_{i}}+\left[\xi I_{t_{i}}^{S}-\gamma Q_{t_{i}}\right]\Delta t\\ H_{t_{i+1}} & = & H_{t_{i}}+\left[q\gamma Q_{t_{i}}-\left(\left(1-x\right)\alpha_{R}+x\alpha_{D}\right)H_{t_{i}}\right]\Delta t\\ R_{t_{i+1}} & = & R_{t_{i}}+\left[\nu \omega I_{t_{i}}^{A}+\left(1-q\right)\gamma Q_{t_{i}}+\left(1-x\right)\alpha_{R}H_{t_{i}}\right]\Delta t\\ D_{t_{i+1}} & = & D_{t_{i}}+\left[x\alpha_{D}H_{t_{i}}\right]\Delta t. \end{array}$$

## 3 Computational results from the statistical data: A case study from the city of Bogotá

In this section, we discuss the stochastic dynamics with environmental noises for the COVID-19 epidemic in Bogotá city. The data used in this article for the simulations, parameter adjustment, and analysis was selected from the publicly available database of the National Institute of Health of Colombia (or in Spanish: Instituto Nacional de Salud, INS) (Instituto Nacional de Salud de Colombia, 2021). This data source provides daily case numbers for both symptomatic and asymptomatic infected individuals without making a clear distinction between the two conditions. It also gives the report of recovered and deceased individuals. However, given that the selected epidemiological data often had irregularities, data cleaning was performed. Subsequently, the new database was filtered with respect to the dates of the analysis. In addition, it is necessary to have the total number of inhabitants in the city of Bogotá D.C. for the mathematical modelling. The population projection of the National Administrative Department of Statistics (or in Spanish: Departamento Administrativo Nacional de Estadística, DANE) (Administrativo Nacional de Estadística, 2018), estimates that for 2020 the total population in the respective city is 7743 955 persons. Finally, the entire methodological procedure was developed in Python 3.7.

**Fig. 2:**
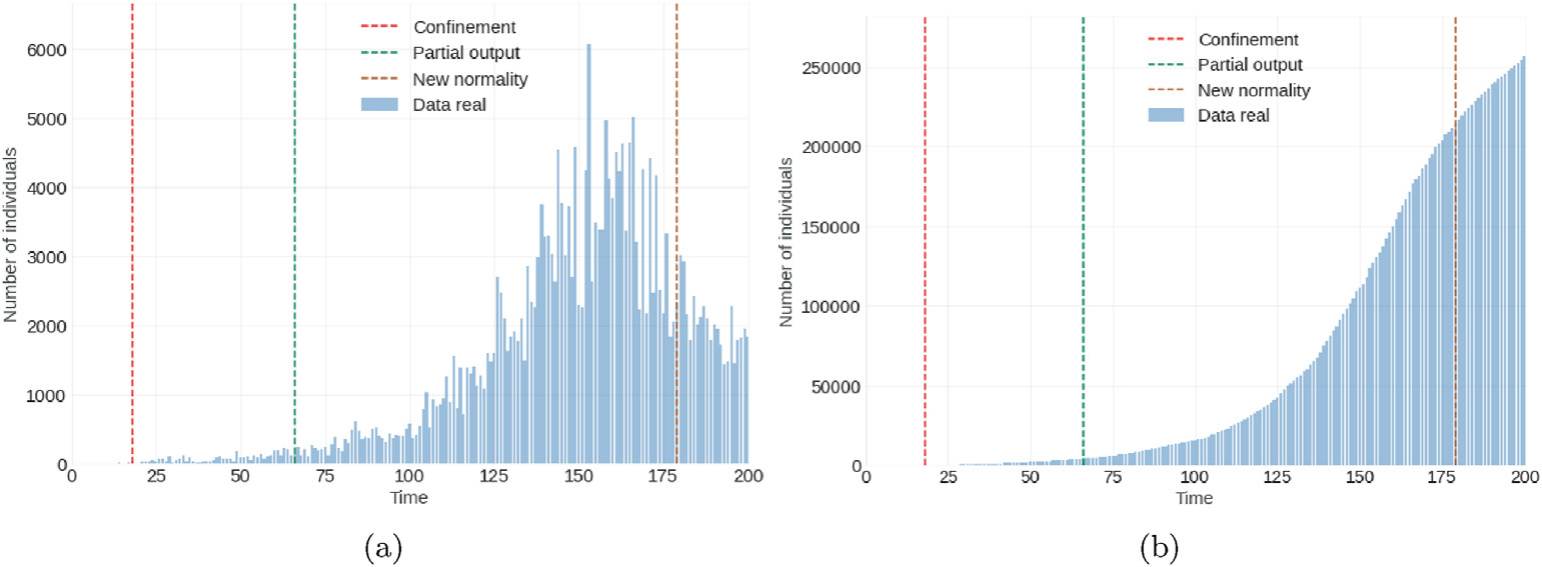
Graph (a) on left denotes number of infected individuals(incidence); Graph (b) on right denotes cumulative number of infected individuals.

**Fig. 3:**
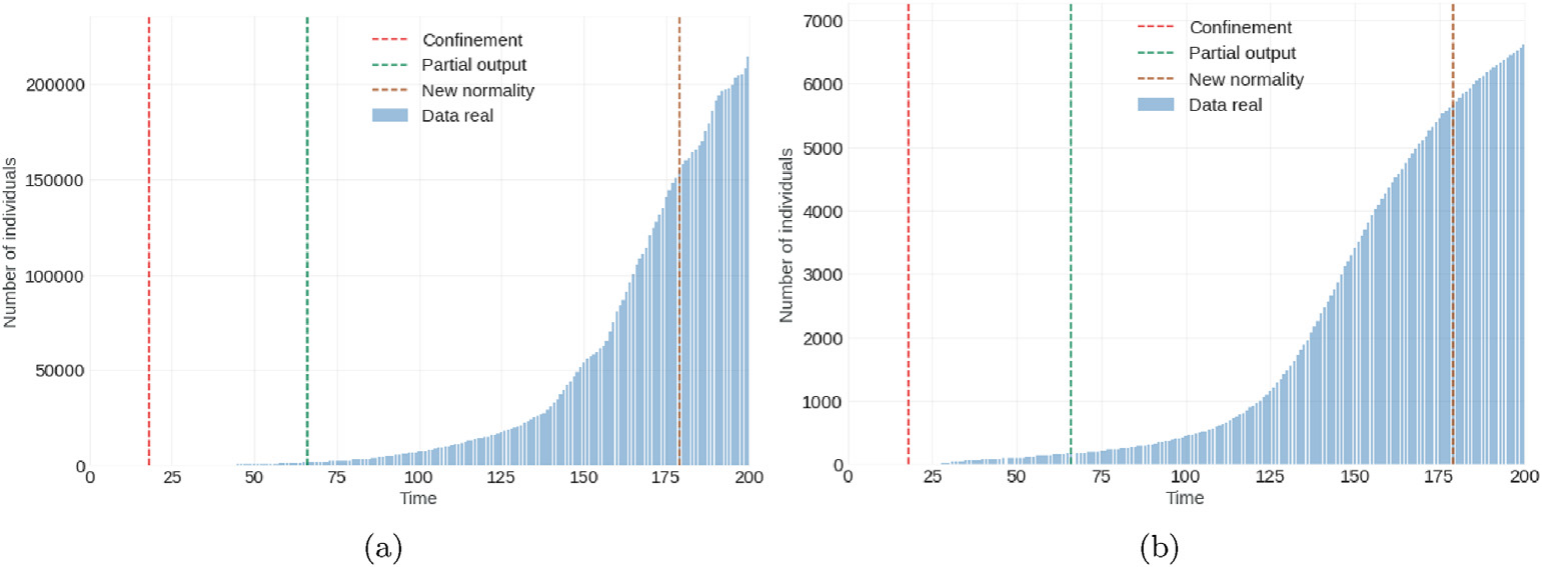
Graph (a) on the left denotes cumulative number of individuals recovered; Graph (b) on the right denotes cumulative number of deceased individuals.

For the proposed model (1) model, we have taken into account the first 200 days of the pandemic in the city of Bogota. The first contagion report occurred on March 6, 2020 (Ministerio de Salud y Protección Social, 2020) (*day 1*) and the end date of the analysis was set for September 22, 2020 (*day 200*). The calibration of the model was carried out with the infected, recovered, and deceased data of the first 100 days, that is, from March 6, 2020, to June 14, 2020 (*day 100*). It is planned for the period from June 15, 2020 to September 22, 2020. The lockdown began on March 24, 2020 (Presidencia de la Repúblicade Colombia, 2020a, 2020b) (*day 18*) and the partial opening began on May 11, 2020 (Presidencia de la Repúblicade Colombia, 2020a, 2020b) (*day 66*). By September 22, 2020, the following was reported in Colombia: 777 537 infected, 650 801 recovered, 24 570 deaths, and 3460 714 processed samples. And for the city of Bogotá D.C, the following was reported: 256,162 infected, 213,956 recovered, and 6,610 deaths (see Table 2). Regarding the daily report of infected in the same city, its maximum value or peak occurred around August 6, 2020, with 6068 infected cases (see Fig. 2, Fig. 3).

**Table 2:** Statistical description of the data for the city of Bogotá D.C

	Infected	Accumulated infected	Recovered	Accumulated recovered	Dead	Accumulated dead
**Mean**	1300.213 198	65 121.263 959	1138.063 830	42 899.111 702	36.927 374	1972.854 749
**Std**	1419.070 253	83 781.640 668	1593.048 383	62 834.182 549	35.517 685	2266.236 273
**Min**	1.000 000	1.000 000	1.000 000	1.000 000	1.000 000	1.000 000
**25%**	112.000 000	2390.000 000	61.250 000	1309.750 000	5.000 000	172.500 000
**50%**	552.000 000	16 853.000 000	372.500 000	9367.000 000	25.000 000	639.000 000
**75%**	2174.000 000	113 841.000 000	1734.500 000	58 568.250 000	65.500 000	3965.000 000
**Max**	6068.000 000	256 142.000 000	7354.000 000	213 956.000 000	122.000 000	6610.000 000

### 3.1 Calibration of the model

The calibration of the proposed model is carried out in order to find a set of parameters so that the model has a good description of the behavior of the system dynamics, i.e. so that the model fits the data as well as possible and allows us to make the desired projections. The search for the parameters can be carried out by means of different optimization algorithms. The non-linear least squares method of trust region was the one chosen (see (Sáez and Rittmann, 1992)), which will be explained later on. Subsequently, the predictions of the model are compared with the real data available for the city of Bogotá.

It is necessary to find the values of the parameters that fit the model according to the available data and satisfy a certain set of constraints to ensure global convergence and consistency of the parameters. We present the nonlinear least squares optimization method of trust region described in (Kloeden and Platen, 1992) for the adjustment of the parameters of the proposed model. Let *θ* = (*θ*_1_, *…*, *θ*_*m*_) be a multidimensional parameter with a neighborhood defined by $B_{\Delta k}\left(p\right)=\left\{\theta_{k}\in \mathbb{R}:||p||\leq \Delta k\right\}$ for 1 ≤ *k* ≤ *m*, where Δ*k* is the radius of the trusted region. Consider $f:R^{n}\to R$ a function that depends on the parameter *θ*.

Given some points (*x*_1_, *y*_1_)⋯(*x*_*n*_, *y*_*n*_) the residual sum of squares is defined as:

(10) $$g\left(\theta \right)=||y-f\left(x;\theta \right)|{|}_{2}^{2}=||R\left(\theta \right)|{|}_{2}^{2}$$

where *g*(*θ*) is the objective function. The information from the objective function in the trust region methods is used to form a model *m*_*k*_ (7), which, near the current point *θ*_*k*_, have as much as possible a behavior similar to that of the objective function.

(11) $$m_{k}\left(p\right)=||R_{k}\left(\theta_{k}\right)+J_{k}p|{|}_{2}^{2}$$

where the Hessian is ▽^2^*g* ≈ *J*^*T*^*J*. Since the model may not be a good approximation of the objective function, when taking points *θ* away from *θ*_*k*_, the search for the minimum *m*_*k*_ that must be restricted to points at *B*_Δ*k*_. So, in each iteration of this methodology, the following sub-problem is solved;

$$\underset{p}{\min\;}m_{k}\left(p\right),$$

subject to ||p||≤Δk.

Given the data on infected, recovered, and deceased, and the information on the parameters obtained from a meta-analysis of the epidemiological and health entities in charge of Bogotá, the parameters of the model were fitted with the non-linear least squares procedure using the trust region algorithm. In Table 3 and Table 4, we can observe the fixed values and those obtained from the model adjustment respectively, and also in the references column we can find the literature, which helped us to establish the fixed and initial values to carry out the optimization process. Note that the standard error for the parameters *α*_*D*_ and *x* are too high, which possibly indicate that there is too much variability of the number of deaths after COVID-19 hospitalized them: there could be days with few casualties and others with too many deaths after being hospitalized by COVID-19.

**Table 3:** Adjusted *drift* parameters.

Parameter	Value	Units	Standard error	Bounds	Reference
*β*	1.063 147	day	2.965 016	[0, 1.4]	de-Camino-Beck (2020); Kumari et al. (2020)
*ω*	0.476 190	*days*^−1^	13.234 271	[1/10, 1/2.1]	Mejía Becerra (2020); Imperial College London (2020)
*ξ*	0.200 000	*days*^−1^	1.614 248	[1/2, 1]	Harvard (2020); Belkhiria and Nascimento (2020)
*α*_*R*_	0.105 263	*days*^−1^	6.379 961	[1/12, 1/9.5]	Jafari. et al. (2021)
*α*_*D*_	0.131 579	*days*^−1^	4.1 526E+2	[1/14, 1/7.6]	Jafari. et al. (2021)
*ν*	0.731 120	–	12.446 559	(0, 1)	Imperial College London (2020)
*x*	0.900 000	–	3.1 375E+3	(0, 1)	Imperial College London (2020)

**Table 4:** Fixed parameters.

Parameter	Value	Units	Reference
*κ*	1/4.6	*days*^−1^	Mejía Becerra (2020); Imperial College London (2020)
*γ*	1/8	*days*^−1^	(Harvard, 2020), (Belkhiria & Nascimento., 2020)
*p*	0.100	–	Mejía Becerra (2020); He et al. (2021)
*q*	0.190	–	Ohajunwa et al. (2020); Belkhiria and Nascimento (2020)
*a*	0.934	–	Assumption
*b*	0.011	–	Assumption
*ε*	5.000	–	Assumption
*t*_*a*_	18	*days*	Presidencia de la Repúblicade Colombia (2020a, 2020b)
*t*_*b*_	66	*days*	Presidencia de la Repúblicade Colombia (2020a, 2020b)

### 3.2 Model projections

**Fig. 4:**
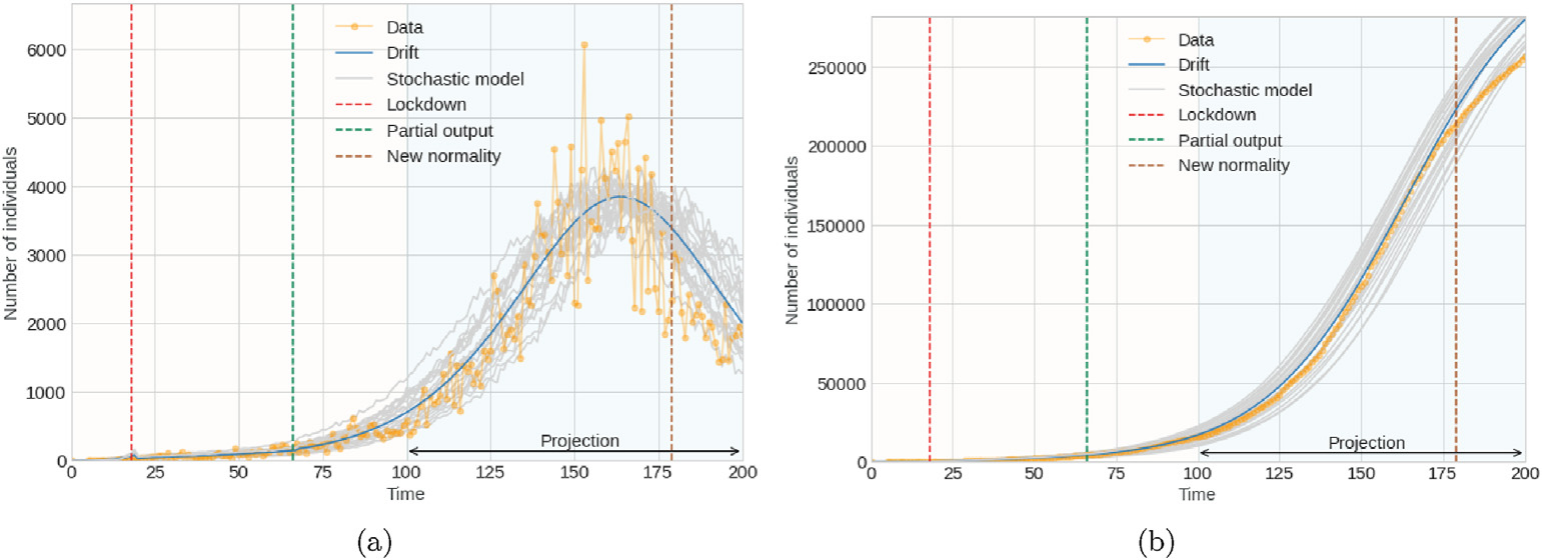
Graph (a) on left denotes number of infected individuals (incidence); Graph (b) on right denotes cumulative number of infected individuals.

**Fig. 5:**
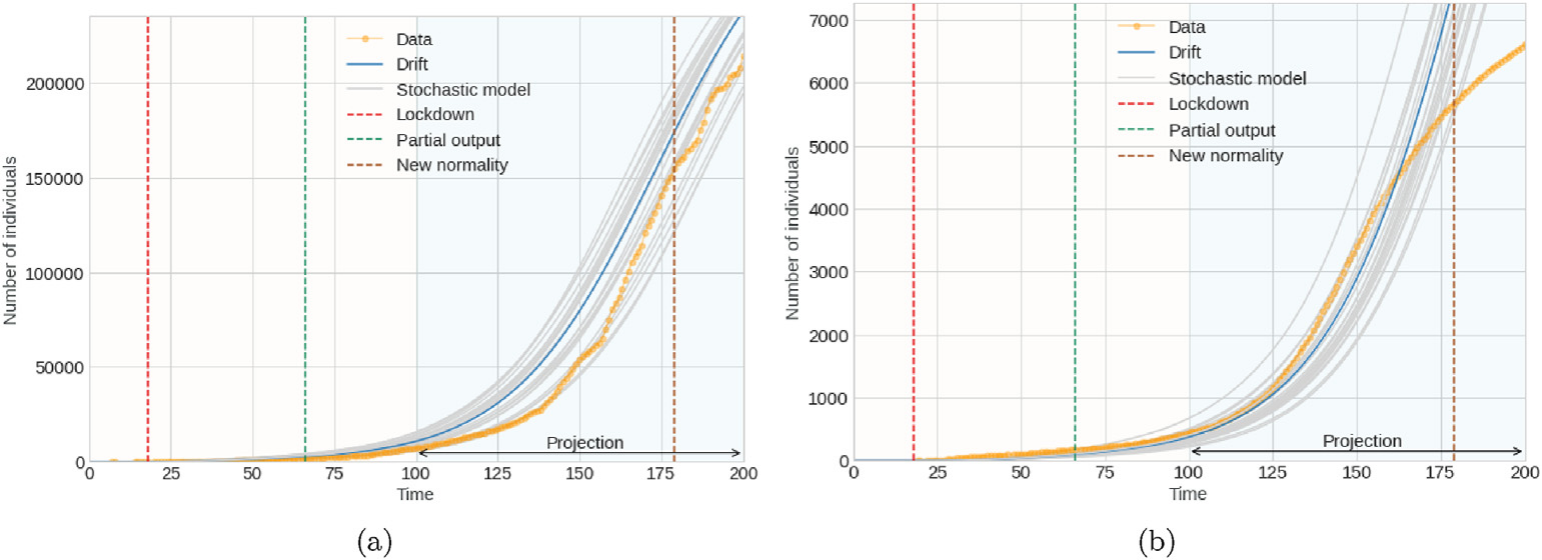
Graph (a) on left denotes cumulative number of individuals recovered; Graph (b) on right denotes cumulative number of deceased individuals.

Fig. 4 and Fig. 5 generally illustrate the contrast between the real dynamics and the projections of the proposed model in the first mitigation measures for the pandemic for the SARS-CoV-2 coronavirus. Fig. 4a shows the analysis of the daily report of individuals infected by the virus, that is, the incidence of infected. In contrast, Fig. 4b shows the analysis of the number of accumulated infected. Fig. 5a and b represents the analysis of accumulated recovered and accumulated deceased individuals, respectively.

In the following figures, the actual data is represented by the yellow line. The gray lines are the 20 simulations represents results from the stochastic model subject to parameter adjustment by the trust region method, and the blue line is the drift of the model. Likewise, the graphs specify the analysis period, that is, the period in which the parameters were projected and adjusted. They also indicate the periods of confinement and partial opening.

The mean square logarithmic error (MSLE) is calculated as an evaluation measure of the proposed stochastic model. The MSLE only considers the relative difference between the actual and the projected value. This measure treats minor differences between small actual and forecasted values, roughly the same way as significant differences between large actual and forecasted values. The cause of errors is significantly penalized than small ones in those cases where the target value range is large. So, since the drift is the stochastic mean, when evaluated against the actual data with the MSLE measure, this gives a cursory idea of how reliable the model is. For the projections of the incidence of infected, an MSLE of 0.090 is obtained, and for the accumulated infected, an MSLE of 0.005, while for the predictions of accumulated recovered, an MSLE of 0.157 and for accumulated deaths an MSLE of 0.062. Therefore, both the graphs and the superficial or general measurement of the MSLE model infer that the projections of the recovered are not very favorable compared to the other cases.

**Fig. 6:**
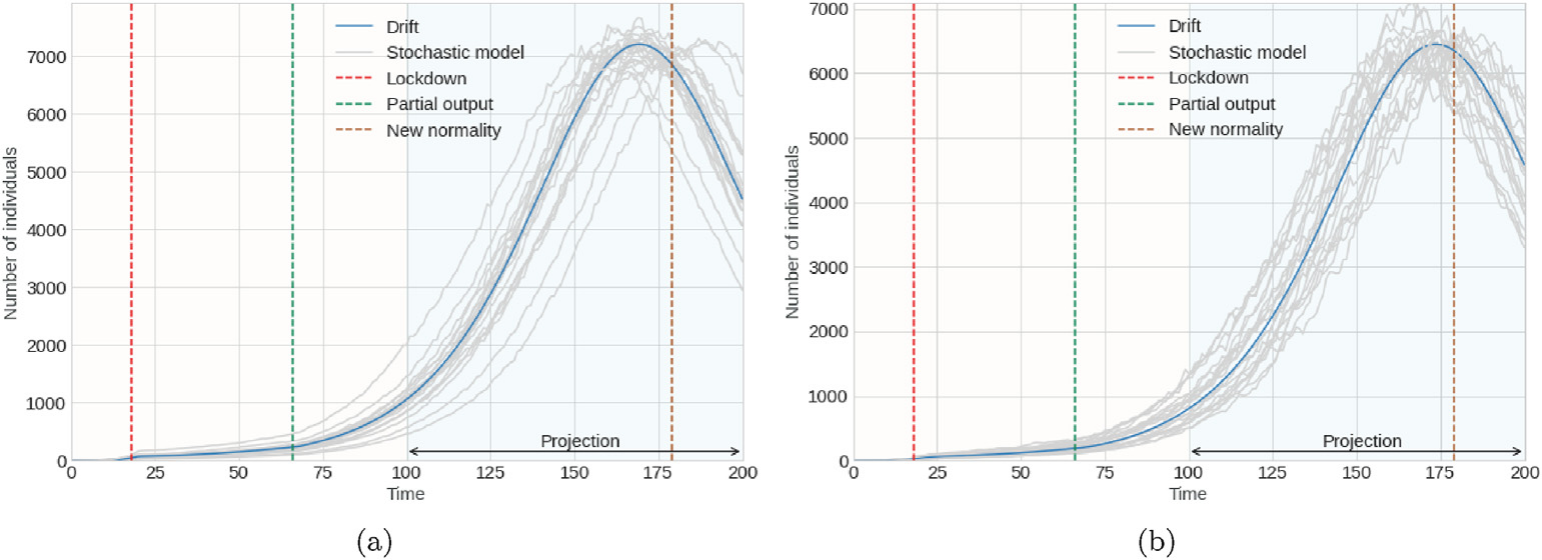
Graph (a) on left denotes number of asymptomatic infected individuals (prevalence); Graph (b) on right denotes number of symptomatic infected individuals (prevalence).

**Fig. 7:**
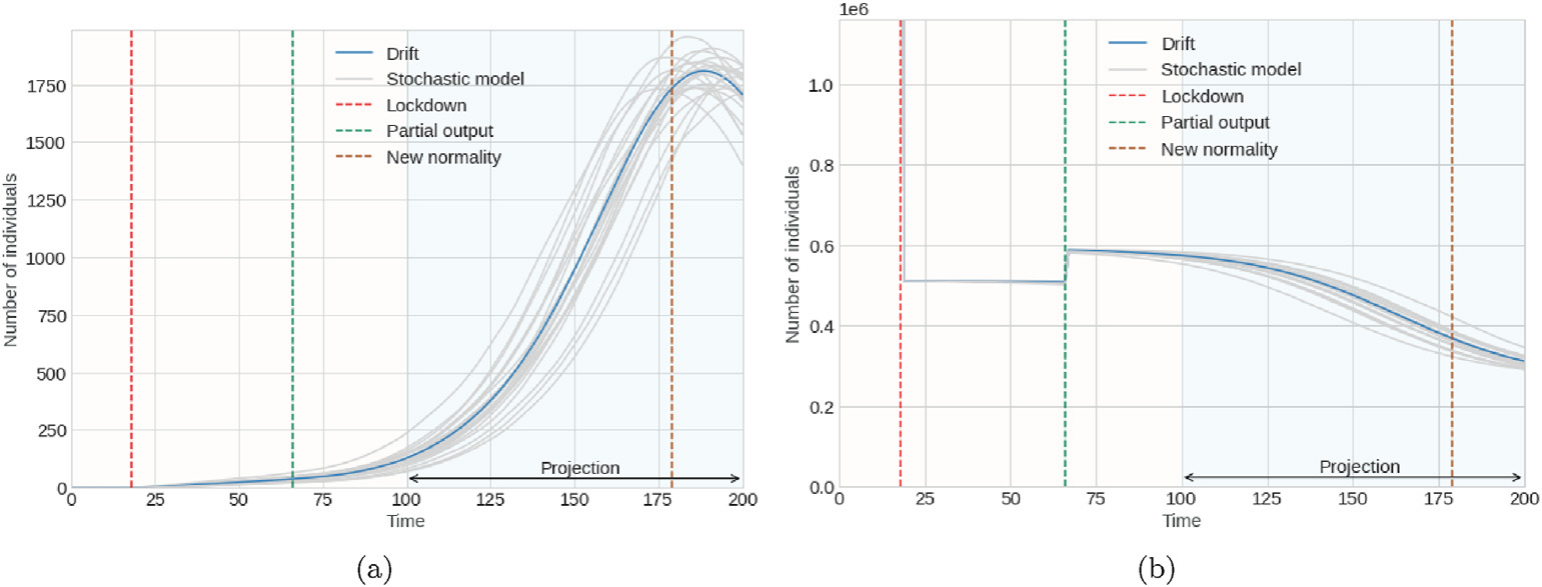
Graph (a) on left denotes number of people hospitalized (prevalence); Graph (b) on right denotes number of susceptible people.

Fig. 6 and Fig. 7 are not contrasting graphs like the previous ones. One reason could be the difficulty of obtaining the prevalence data for the cases mentioned below. However, as can be seen, the graphs represent the 20 simulations of the stochastic model, the drift of the model given the adjustment of the parameters and with intensity constants *σ*_1_ = 0.2 and *σ*_2_ = 0.4 and the dates of the mitigation strategies chosen by the national government. Fig. 6a and b show possible projections of the stochastic model for asymptomatic and symptomatic infected individuals, respectively. In contrast, Fig. 7a represent simulations of the prevalence of hospitalized individuals, and Fig. 7b represents these changes in the stochastic dynamics of susceptible individuals.

In our model, we obtain the basic reproduction number for the proposed model (theoretically calculated in (Ohajunwa et al., 2020)) given by

$$R_{0}^{\text{Cov}}=\beta \left(\frac{2p}{\xi }+\frac{1-p}{\omega }+\frac{\left(1-\upsilon )(1-p\right)}{\xi }\right)$$

Also, from the parameters given in Table 4, we note that the basic reproduction number for COVID-19 in Bogotá has normal distribution with median $R_{0}^{\text{Cov}}=4.34$ and variance 1.79 (standard deviation 1.34) given by:

$$\frac{\sigma_{1}^{2}{\left({\left(1-\upsilon \right)}^{2}\left(1-p\right)+p\right)}^{2}}{2\xi^{3}}+\frac{\sigma_{2}^{2}p^{2}}{2\xi^{3}}+\frac{\sigma_{2}^{2}{\left(1-p\right)}^{2}}{2\omega^{3}}+\frac{2\sigma_{2}^{2}p\left(1-p\right)}{\left(\xi +\omega \right)\xi \omega }$$

suggesting that COVID-19 could be an *endemic* for Bogotá. This may also be due to the *μ* variant B.1.621 identified in Colombia and recognized by WHO in the list of variants that reported 25% of new incidence cases in Colombia (see (Khan Burki, 2021; Shultzet al., 2021)).

## 4 Conclusion

In this work, a new stochastic epidemiological model is proposed that accounts for randomness in the dynamics of COVID-19. The model provides a perspective of the possible scope for the spread of COVID-19 with random behaviors in the population. We were able to show the importance of including stochasticity within the population that models the real data from Colombian city of Bogotá. Our computational simulations suggest that the proposed model is reliable and robust in projecting the number of infected, recovered and deceased individuals during interventions such as confinement and partial opening. The results from this work can be used to inform data-driven decision making for improving public health in Colombia.

While the model seems to capture the data from the Colombian city of Bogotá well, it is potentially limited by the lack of prevalence data corresponding to registration of individuals who present a certain characteristic of the disease over a period of time, including susceptible, exposed, hospitalized and quarantined sub-populations, as well as the complete registry of the data of asymptomatic infected. The proposed model explains the containment of the pandemic by strict lock-down and decline in number of incidence cases and death. This work also opens a way to explore newer compartment models one can take into account including other factors such as migrations, demographics, spatial and vaccination effects. One can also compare the proposed model against reliable data from another country. Another potential area of research includes using these models in conjunction with machine learning and deep learning to obtain optimal parameters. Another area that can be considered is to study to what extent the results can be generalized to the overall population and across different geographical areas as well as patterns of contact between and within groups and in different social settings. All these will be considered in forthcoming papers.

## Availability of data and material

Not applicable. All data generated or analyzed during this study are included in this manuscript.

## Funding

This work was financial support by Directorate-Bogotá campus (10.13039/501100002945DIB), Universidad Nacional de Colombia under the project No. 50803.

## Declaration of competing interest

The authors declare that they have no known competing financial interests or personal relationships that could have appeared to influence the work reported in this paper.
